# The NADPH Oxidase Isoform 1 Contributes to Angiotensin II-Mediated DNA Damage in the Kidney

**DOI:** 10.3390/antiox9070586

**Published:** 2020-07-05

**Authors:** Anna Zimnol, Nora Spicker, Ronja Balhorn, Katrin Schröder, Nicole Schupp

**Affiliations:** 1 Institute of Toxicology, Medical Faculty, University of Düsseldorf, 40225 Düsseldorf, Germany; anna_zimnol@web.de (A.Z.); nora.lea@hotmail.com (N.S.); balhorn.ronja@hhu.de (R.B.); 2 Institute for Cardiovascular Physiology, Goethe-University, 60596 Frankfurt, Germany; schroeder@vrc.uni-frankfurt.de

**Keywords:** hypertension, NADPH oxidase, DNA damage, kidney function

## Abstract

In higher concentrations, the blood pressure regulating hormone angiotensin II leads to vasoconstriction, hypertension, and oxidative stress by activating NADPH oxidases which are a major enzymatic source of reactive oxygen species (ROS). With the help of knockout animals, the impact of the three predominant NADPH oxidases present in the kidney, i.e., Nox1, Nox2 and Nox4 on angiotensin II-induced oxidative damage was studied. Male wildtype (WT) C57BL/6 mice, Nox1-, Nox2- and Nox4-deficient mice were equipped with osmotic minipumps, delivering either vehicle (PBS) or angiotensin II, for 28 days. Angiotensin II increased blood pressure and urinary albumin levels significantly in all treated mouse strains. In Nox1 knockout mice these increases were significantly lower than in WT, or Nox2 knockout mice. In WT mice, angiotensin II also raised systemic oxidative stress, ROS formation and DNA lesions in the kidney. A local significantly increased ROS production was also found in Nox2 and Nox4 knockout mice but not in Nox1 knockout mice who further had significantly lower systemic oxidative stress and DNA damage than WT animals. Nox2 and Nox4 knockout mice had increased basal DNA damage, concealing possible angiotensin II-induced increases. In conclusion, in the kidney, Nox1 seemed to play a role in angiotensin II-induced DNA damage.

## 1. Introduction

Hypertension increases heart attacks, strokes, and chronic kidney disease. Additionally, hypertensive patients exhibit an increased incidence of cancer, especially renal cell cancer, for which hypertension is an established risk factor [1,2,3]. As a prerequisite for cancer development, mutations occur in cells which often arise from DNA damage. Therefore, as a possible molecular mechanism for the increased cancer risk due to hypertension, we have been studying genotoxic effects of the hormone system that controls blood pressure, the renin angiotensin aldosterone system (RAAS), which is often deregulated in hypertensive patients [4,5]. In vitro, ex vivo in mouse kidneys and in vivo in animal models of hypertension, we have found angiotensin II to cause DNA lesions [6,7,8]. Recently, we have proven that chronic angiotensin II excess also leads to permanent mutations in kidney cell DNA of rats [9].

Reactive oxygen species (ROS) play a major role in angiotensin II-induced DNA damage since it can be prevented in vitro and in vivo by concomitant treatment with antioxidants [10,11]. Angiotensin II is known to induce the production of reactive oxygen species (ROS) via the activation of NADPH oxidases [12,13]. NADPH oxidases are multimeric enzymes generating superoxide anion radicals or hydrogen peroxide out of oxygen while reducing NADPH. Seven isoforms of the catalytic Nox subunit have been identified in mammalian cells, Nox1–5 and Duox1/2 [14]. In the kidney Nox1, -2, and -4 are expressed [15]. Silencing the isoform Nox4 in a human kidney cell line prevents angiotensin II-induced DNA damage, which could not be achieved by silencing Nox2 [10].

To know which of the Nox isoforms is able to generate genomic damage possibly can help to elucidate mechanisms of increased cancer incidence and mortality in chronic diseases such as hypertension, diabetes, or chronic kidney disease [16]. To identify the Nox isoform responsible for the formation of angiotensin II-induced DNA lesions, angiotensin II-mediated hypertension was induced in wildtype mice and in mice deficient in one of the three NADPH oxidase catalytic subunits Nox1, -2, or -4.

## 2. Materials and Methods

### 2.1. Animals and Animal Treatment

The experiments reported here were conducted in four parts (found under Section 2.1.1, Section 2.1.2 and Section 2.1.3), each with 5–6 wildtype animals in a control and a treatment group additional to the knockout animals listed below, with the Nox4 knockout mice experiments conducted in two subsequent experiments. In all four parts, the animals were treated exactly in the same manner, as described here.

Out of forty-two male wildtype C57BL/6J (WT) mice (Janvier, Le Genest Saint Isle, France), eight groups with five to six animals were formed. The mice were housed in animal facilities at the University of Würzburg or the University of Düsseldorf, under pathogen-free conditions with a room temperature of 20 ± 2 °C, a 12 h light/dark cycle, and free access to standard food and water. At the age of 14 to 18 weeks, the animals were equipped with osmotic mini pumps (Alzet, Model 1004; Durect Corporation, Cupertino, CA, USA) as recommended by Durect Corporation (www.alzet.com/resources/downloads/#surgical-sheets) under general anesthesia (ketamine 90 mg/kg, Pfizer, Berlin, Germany and xylazine 6 mg/kg i.m., Bayer Vital, Leverkusen, Germany). Preemptive analgesia was provided with 45 mg/kg metamizol (MSD Animal Health/Intervet International GmbH, Unterschleißheim, Germany). Four groups received angiotensin II (Calbiochem, Darmstadt, Germany) in a concentration of 600 ng/kg min for 28 days, a concentration causing hypertension, oxidative stress, and DNA damage in the kidney over the treatment time as reported before [11,17], while the other four groups received the solvent control PBS via the pump. Four mice of the angiotensin II-treated groups were lost due to infection or pump malfunctioning.

#### 2.1.1. Part 1: Nox1 Knockout Mice

Sixteen male NADPH oxidase isoform 1 (Nox1) knockout mice (B6.129 × 1-Nox1^tm1Kkr^/J, The Jackson Laboratory, Bar Harbor, ME, USA), furthermore abbreviated as Nox1^y/−^, were randomly distributed to two groups. As described above, one group received 600 ng angiotensin II/kg min for 28 days, while the other group received the solvent control PBS via osmotic mini pumps. One mouse of the angiotensin II-treated group was lost due to infection or pump malfunctioning.

#### 2.1.2. Part 2: Nox2 Knockout Mice

Sixteen male NADPH oxidase isoform 2 (Nox2) knockout mice (B6.129S6-Cybb^tm1Din^/J, The Jackson Laboratory, Bar Harbor, ME, USA), furthermore abbreviated as Nox2^y/−^, were randomly distributed to two groups. As described above, one group received 600 ng angiotensin II/kg min for 28 days, while the other group received the solvent control PBS via osmotic mini pumps. Two mice of the control and one mouse of the angiotensin II-treated group were lost due to infection or pump malfunctioning.

#### 2.1.3. Parts 3 and 4: Nox4 Knockout Mice

Twenty male NADPH oxidase isoform 4 (Nox4) knockout mice (Nox4^−/−^, kindly donated by Prof. Schröder, Vascular Research Centre, University of Frankfurt, Frankfurt, Germany), furthermore abbreviated as Nox4^−/−^, were randomly distributed to two groups. As described above, one group received 600 ng angiotensin II/kg min for 28 days, while the other group received the solvent control PBS via osmotic mini pumps.

Blood pressure was measured via the noninvasive tail-cuff method twice a week (Visitech Systems, Apex, NC, USA). For this, mice were placed in a restrainer, the tail was inserted in the tail cuff, which after being inflated, by transmission photoplethysmography recorded the amount of blood in the tail vessels, which dilated during the systole and constricted during the diastole (www.visitechsystems.com). Two weeks prior to implantation of the minipumps, mice were trained three times a week for upcoming blood pressure measurements. At the beginning and at the end of the experiment, mice were put in metabolic cages for 22 h to collect urine samples to assess the renal function of the animals. After 4 weeks of treatment, animals were deeply anesthesized (ketamine 120 mg/kg and xylazine 8 mg/kg i.m.) and the organs were perfused with ice-cold Deltadex 40 (AlleMan Pharma, Rimbach, Germany) containing 1% procainhydrochloride (Steigerwald, Darmstadt, Germany), followed by perfusion with ice-cold 0.9% NaCl solution (Fresenius Kabi, Bad Homburg, Germany). Kidneys and hearts were removed, weighed, divided, and were either embedded in paraffin or shock frozen in liquid nitrogen. All animal experiments were performed in accordance with the European Community guidelines for the use of experimental animals and with the German law for the protection of animals (55.2-2531.01-56/11 and 84-02.04.2014.A383). The investigation conformed to the “Guide for the Care and Use of Laboratory Animals” published by the U.S. National Institutes of Health (NIH Publication No. 85-23, revised 1996).

### 2.2. Renal Function

Renal function parameters were assessed by determination of urinary creatinine, serum creatinine, and albumin excretion by commercially available ELISA kits. For quantification of albumin excretion, the Mouse Albumin ELISA Kit (Assay Pro, St. Charles, MO, USA) was used according to the manufacturer’s instructions. Creatinine levels in serum and urine were determined with the Creatinine Urinary/Serum Assay Kit (Cayman Chemical Co., Ann Arbor, MI, USA) according to the protocol provided by the manufacturer. Urea in the serum was determined with the QuantiChromTM Urea Assay Kit (BioAssay Systems, Hayward, CA, USA) according to the protocol provided by the manufacturer.

### 2.3. Histopathology

For histopathological investigation of the kidney, 2 µm paraffin sections were cut and stained with hematoxylin and eosin (HE), periodic acid-Schiff stain (PAS), and Sirius red stain. In the kidneys, the glomerular sclerosis index (GSI), the mesangiolysis index (MSI), the tubulointerstitial sclerosis index (TSI), and the vascular sclerosis index (VSI) were determined, as described in [18].

### 2.4. Measurement of ROS Formation

Reactive oxygen species (ROS) production was measured by staining 5 µm cryosections with 10 µM dihydroethidium (DHE) for 20 min as reported earlier [19]. Pictures of the cells were taken and the cells were quantified by using the cell image analysis software CellProfiler [20] or ImageJ (http://imagej.nih.gov/ij/index.html) within 8 visual fields by measuring grey values of about 800 nuclei each. As a urinary oxidative stress marker, 15-isoprostane F_2t_ was measured with the Urinary Isoprostane ELISA Kit (Oxford Biomedical Research, Oxford, MS, USA) according to the protocol provided by the manufacturer. As a marker of nitrosative stress, 3-nitrotyrosine in serum was measured with the Nitrotyrosine ELISA Kit (StressMarq Biosciences Inc., Victoria, BC, Canada) according to the protocol provided by the manufacturer.

### 2.5. Isolation of Primary Kidney Cells for DNA Strand Break Detection via Comet Assay

Primary kidney cells from the middle of freshly obtained kidney were isolated and subjected to single cell electrophoresis, as described earlier by [17]. For analysis, a fluorescent microscope at 200× magnification and a computer-aided image analysis system (Komet 5, Kinetic Imaging, Bromborough, UK) were used. Fifty cells in total were analyzed per sample. The DNA damage is given as mean tail DNA (percentage of fluorescence in the tail region).

### 2.6. Immunohistochemistry

Kidney sections (2 µm) (RM 2165, Leica, Wetzlar, Germany) were deparaffinized using Roti-Histol (Roth, Karlsruhe, Germany) and ethanol. Antigen retrieval was performed with 0.01 mol/L citrate buffer (pH 6.0) at 95 °C for 15 min. Staining for γ-H2AX-positive cells with the primary antibody (anti-γ-H2AX, Cell Signaling, Herts, UK) was performed as described before [8]. Pictures were taken with a Leica DM750 microscope (Leica Microsystems GmbH, Wetzlar, Germany) at a 200-fold magnification. For quantification of γ-H2AX-positive cells, i.e., cells containing double strand breaks, in the kidney, the percentage of positive cells was assessed by counting about 3000 nuclei per mouse.

Due to technical problems with the paraffin samples of Nox2 knockout animals and their respective wildtype controls, here, γ-H2AX-positive cells were stained on cryo-cuts using a fluorescent method. For this, kidney cryo-cuts (5 µm) were fixed with formaldehyde and permeabilized with 0.2% Nonidet P40. After blocking with goat serum at room temperature for 60 min, the primary antibody (anti-γ-H2AX, Cell Signaling, Herts, UK) was added, diluted 1:200 in Signal Stain (Cell Signaling, Herts, UK), and incubated overnight at 4 °C. Then, a Cy3-conjugated secondary antibody (111-166-045, Jackson ImmunoResearch, West Grove, PA, USA) was added for 45 min. The samples were mounted with Vectashield (H-1200, Vectorlabs, Burlingame, CA, USA). Pictures were taken at a 40× enlargement with an Olympus XM10 camera connected to an Olympus BX43 microscope (Olympus, Hamburg, Germany). Six to eight visual fields with a total of 2500 cells minimum were analyzed with ImageJ, relating the γ-H2AX-positive nuclei to the total number of DAPI-stained nuclei.

### 2.7. Statistics

Data from 5–20 animals are shown as mean ± standard error mean (SEM). Differences between the angiotensin II-treated groups and their respective untreated control were tested for significance using the unpaired two-tailed Student’s *t*-test or with one-way analysis of variance (ANOVA) and subsequent post-hoc two-sided comparisons by Tukey. Additionally, differences between the wildtype animals and the knockout animals were tested with two-way ANOVA and subsequent post-hoc two-sided comparisons by Tukey. A *p* value ≤ 0.05 was considered to be significant.

## 3. Results

### 3.1. Animal Characteristics and Blood Pressure Changes

Treatment with angiotensin II resulted in a significantly lower body weight in all angiotensin II-treated groups except in the Nox1 knockout animals (Table 1). While there was no effect of angiotensin II seen on the relative kidney weight, the hearts of animals of all angiotensin II-treated groups except for the Nox4 knockout group showed a significant gain of weight.

**Figure 1:**
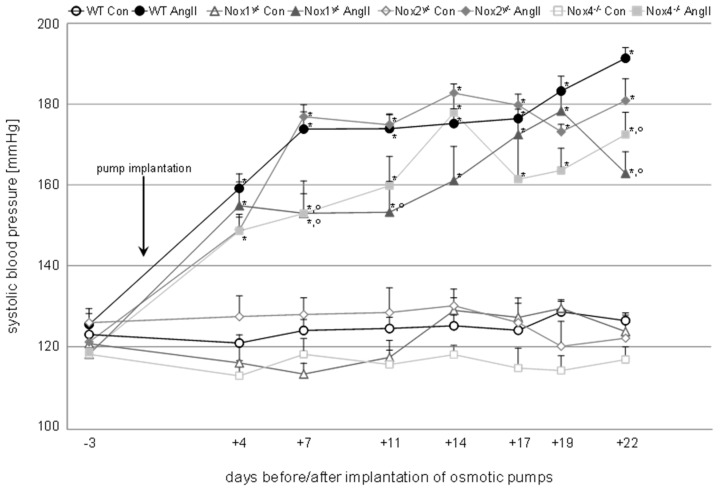
Time course of systolic blood pressure measured in wildtype and knockout animals of the control (Con) and angiotensin II-treated (AngII) groups. Blood pressure was measured at eight time points after habituation to the measurement procedure. The first time point represents the initial systolic blood pressure of the animals before implantation of the osmotic minipumps. After the implantation, blood pressure was measured twice a week. Data are shown as mean + SEM. * *p* ≤ 0.01 vs. the respective control, and ° *p* < 0.05 vs. angiotensin II-treated WT animals analyzed by one-way ANOVA. WT, wildtype; Con, control; AngII, angiotensin II; Nox, NADPH oxidase catalytic subunit.

A significant increase in systolic blood pressure was induced in all angiotensin II-treated animals over three weeks out of the four weeks of treatment time (Table 1, Figure 1). The systolic pressure of Nox1 animals was in three, and that of Nox4 animals in two, out of seven measurements significantly lower than the systolic pressure of WT animals, one of those time points being the end of the treatment (Figure 1). Diastolic blood pressure was significantly increased in all angiotensin II-treated groups except in the Nox1 knockout animals (Table 1).

### 3.2. Renal Function and Morphology

**Figure 2:**
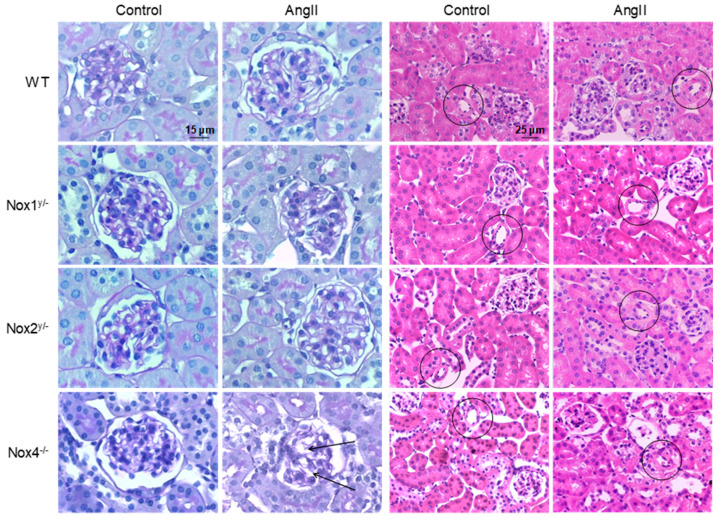
Angiotensin II-induced morphological changes in the kidney. Representative pictures of kidney slices of all animal groups. The two columns on the left (400-fold magnification) show PAS-stained tissue, the arrows point to dilated vessels in the glomerulus, caused by the loss of mesangial matrix. The two columns on the right (200-fold magnification) show HE-stained tissue, the circles highlight small vessels, which have thicker vessel walls in all angiotensin II-treated groups except in Nox1 knockout animals.

There were no differences between angiotensin II-treated animals and their respective controls observed in the histopathological analysis of glomeruli and tubuli (Table 2 and Figure 2). The glomerular sclerosis, mesangiolysis, and the tubulointerstitial sclerosis index were not changed by the treatment, with the exception that mesangiolysis increased in the angiotensin II-treated Nox4 knockout animals. Significant changes were found when studying the renal vasculature. Here, all groups treated with angiotensin II presented increased vascular wall diameters with the exception of the angiotensin II-treated Nox1 animals, whose vascular sclerosis index was significantly lower than that of the angiotensin II-treated wildtype mice (Table 2 and Figure 2).

**Figure 3:**
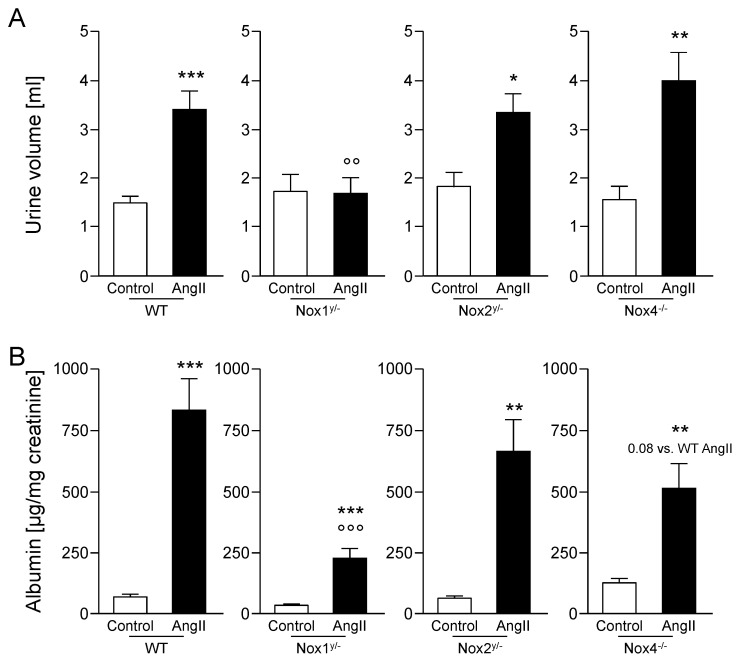
Angiotensin II led to polyuria and albuminuria, which were less pronounced in Nox1 knockout mice. (**A**) Urinary volume; (**B**) Albumin excreted related to creatinine in urine. Data are shown as mean + SEM. * *p* ≤ 0.05, ** *p* < 0.01, *** *p* < 0.001, vs. the respective control animals analyzed by the Student’s *t*-test; °° *p* < 0.01 (F(1.48) = 4.60), °°° *p* < 0.001 (F(1.49) = 10.37) vs. angiotensin II-treated WT animals, analyzed by two-way ANOVA. WT, wildtype; AngII, angiotensin II; Nox, NADPH oxidase catalytic subunit.

The angiotensin II treatment increased water uptake and urinary volume significantly in all groups, except in the Nox1 knockout animals (Table 2 and Figure 3A). The urinary volume of angiotensin II-treated Nox1 knockout mice was significantly lower than the urinary volume of angiotensin II-treated wildtype mice.

There was no effect observed for creatinine clearance (Table 2). Albumin concentration in urine was significantly increased in all animals treated with angiotensin II as compared with the respective control animals (Figure 3B). Albuminuria was also significantly higher in the Nox1 knockout animals, but lowest of all groups, and significantly lower than in the angiotensin II-treated wildtype mice. Also in the angiotensin II-treated Nox4 knockout animals, albuminuria was lower, almost reaching significance as compared with the angiotensin II-treated wildtype mice.

### 3.3. Markers of Oxidative Stress and DNA Damage

**Figure 4:**
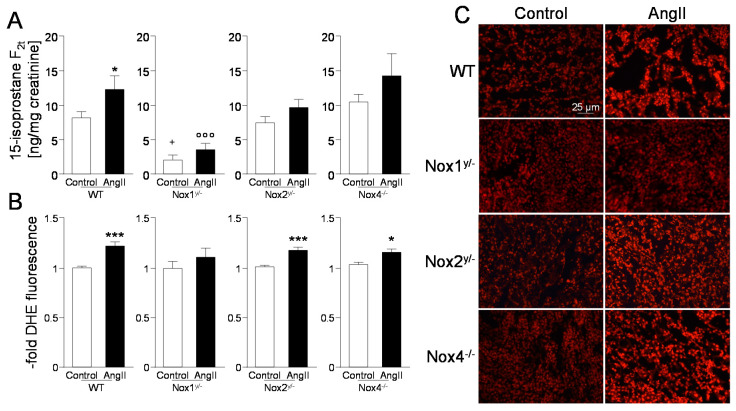
Angiotensin II led to systemic oxidative stress only in wildtype mice, but also to local oxidative stress in Nox2 and Nox4 knockout mice. (**A**) Systemic oxidative stress measured as excretion of 15-isoprostane F_2t_ in urine; (**B**) Local oxidative stress in the kidney measured with the help of the redox sensitive dye dihydroethidium and quantified with the cell image analysis softwares, CellProfiler [20] or ImageJ; (**C**) Representative pictures of DHE stained kidney tissue of all groups (100-fold magnification). Data are shown as mean + SEM. * *p* ≤ 0.05, *** *p* < 0.001, vs. the respective control animals analyzed by the Student’s *t*-test; ^+^ *p* ≤ 0.05 (F(1.39) = 3.97) vs. control WT animals; °°° *p* < 0.001 (F(1.39) = 27.00) vs. angiotensin II-treated WT animals, analyzed by two-way ANOVA. WT, wildtype; AngII, angiotensin II; Nox, NADPH oxidase catalytic subunit.

Systemic oxidative stress was measured as excretion of products of lipid oxidation with 15-isoprostane F_2t_ as the marker substance. While a tendency to higher isoprostane levels was seen in all angiotensin II-treated animals, except in the angiotensin II-treated Nox1 knockout mice, the increase was only significant in the WT mice (Figure 4A). Acute oxidative stress measured with the redox sensitive dye dihydroethidium (DHE) on renal tissue was significantly increased in all angiotensin II-treated mice except in Nox1 knockout mice (Figure 4B,C).

**Figure 5:**
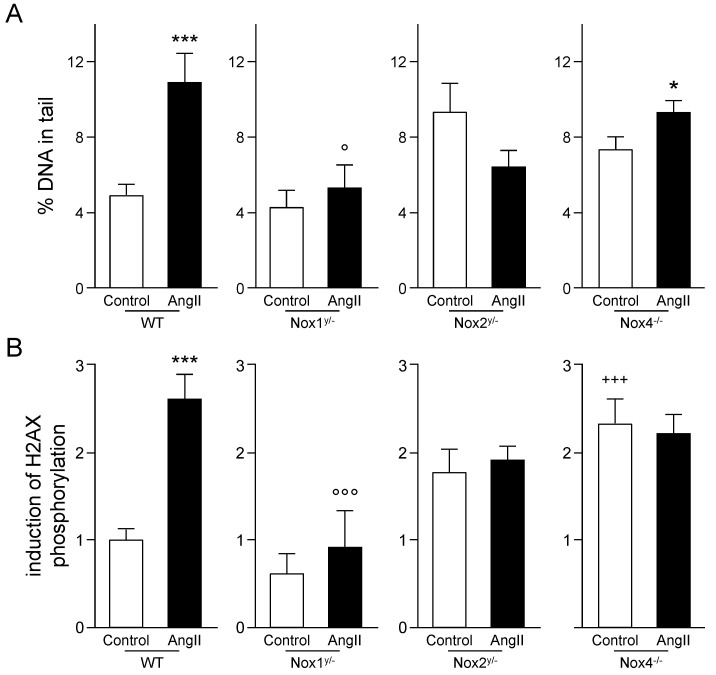
DNA damage caused by angiotensin II. (**A**) DNA damage measured by the comet assay in kidney cells extracted from kidneys at the end of the experiment. Results are shown as percentage of DNA in tail + SEM; (**B**) Quantification of cells positive for phosphorylated H2AX (γ-H2AX) in the cortex of the kidney within eight visual fields after immunohistochemical detection of γ-H2AX on paraffin-embedded or frozen kidney sections with the help of the cell image analysis software CellProfiler [20]. Results are shown as induction of H2AX phosphorylation + SEM. * *p* ≤ 0.05, *** *p* < 0.001, vs. the respective control animals analyzed by the Student’s *t*-test; ^+++^ *p* < 0.001 (F(1.50) = 9.64) vs. WT control animals; and ° *p* ≤ 0.05 (F(1.45) = 5.74, °°° *p* < 0.001 (F(1.49) = 13.89) vs. angiotensin II-treated WT animals, analyzed by two-way ANOVA.

DNA damage induced by angiotensin II in the kidney of the four mouse strains was analyzed using two methods. The comet assay, detecting acute structural DNA damage which mainly consists of DNA single-strand breaks and abasic sites, showed significantly increased damage in freshly isolated kidney cells of WT and Nox4 knockout animals treated with angiotensin II (Figure 5A). No significant change induced by angiotensin II was seen in kidney cells from Nox1 and Nox2 knockout animals. Basal DNA damage was higher in kidneys from Nox2 and Nox4 knockout mice than in kidneys from WT mice, without reaching significance.

**Figure 6:**
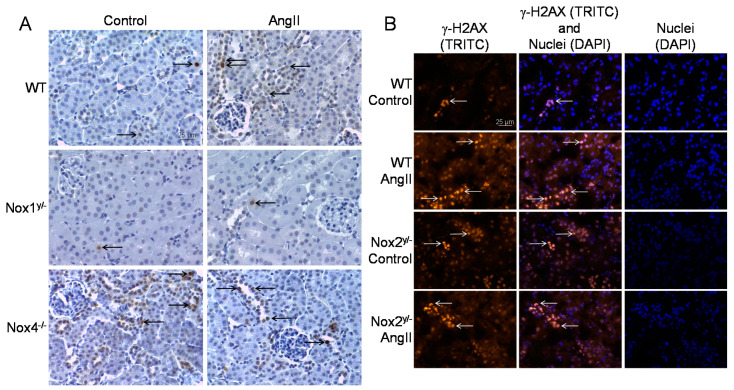
DNA damage in the kidneys of wildtype and Nox knockout animals. (**A**) Representative pictures of the immunohistochemical staining of paraffin-embedded kidney sections of animals of the wildtype (WT), the Nox1 and Nox4 knockout group for the surrogate marker of DNA double-strand breaks, phosphorylated H2AX (γ-H2AX); (**B**) Representative pictures of the immunofluorescent staining of frozen kidney sections of animals of the wildtype (WT) and Nox2 knockout group for the surrogate marker of DNA double-strand breaks, phosphorylated H2AX (γ-H2AX) with 200-fold magnification, the fluorescence is presented in pseudo-colors.

The second method employed was the immunochemical detection and quantification of the phosphorylated histone H2AX (γ-H2AX), which is a surrogate marker for DNA double-strand breaks. γ-H2AX was significantly increased by angiotensin II only in WT kidneys (see Figure 5B and Figure 6). Kidney tissue from Nox1 knockout mice showed no change in H2AX phosphorylation after angiotensin II treatment. Kidneys from Nox2 and Nox4 knockout mice presented an already increased basal H2AX phosphorylation already without angiotensin II treatment. This was significantly higher in the Nox4 knockout strain than in the WT control.

## 4. Discussion

To identify the NADPH oxidase isoform responsible for angiotensin II-induced oxidative damage in hypertensive animals, knockout animals lacking the catalytic subunits of the three most abundant NADPH oxidase catalytic subunit isoforms 1, 2, and 4 were treated with angiotensin II for four weeks. Angiotensin II increased the blood pressure significantly in wildtype and all knockout animals. Although, in angiotensin II-treated knockout animals that were lacking the isoforms 1 and 4, the blood pressure was at some time points lower than that of wildtype animals, this was not the case for isoform 2 knockout animals. There have been contradicting reports with respect to the influence of Nox isoform knockouts on blood pressure upon angiotensin II infusion or RAAS activation. Some working groups have reported no difference in blood pressure values between wildtype animals and knockout animals (Nox1 [21], Nox2 [22,23], and Nox4 [24]). Others have reported lower baseline blood pressures and lower pressure increases after angiotensin II infusion (Nox1 [22,25], Nox2 [26], and Nox4 [27]).

Except in Nox1 knockout animals, angiotensin II treatment caused higher water consumption and increased urine excretion. Unfortunately, no information on these parameters could be found in other publications using Nox1 knockout mice. Rac1-stimulated NADPH oxidases, which comprise Nox1, Nox2, and probably also Nox3 [28], seem to be involved in dipsogenic effects of angiotensin II, because expression of dominant-negative Rac1 abolished the drinking response [29]. Brain specific deletion of Nox2 also significantly reduced thirst and, thereby, water intake in mice to a level far below the control level, leading to the conclusion by the authors that Nox2 was solely responsible for the angiotensin II-induced drinking response [30]. We did not see an impact of the whole body Nox2 knockout on water intake, in our study, but we did not monitor the actual efficiency of the knockout in the brain. Furthermore, the authors [30] measured acute thirst induced 30 min after angiotensin II injection rather than longtime effects as we did in our study. The angiotensin II-treated Nox1 knockout mice in the present study, had the same water intake and urine output as the control and wildtype control animals, after 28 days of angiotensin II infusion, although their blood pressure increased. This hints to an important role of Nox1 in angiotensin II-induced thirst.

Morphologically, all angiotensin II-treated animals, with the exception of the Nox1 knockout animals, showed a thickening of small vessels in the kidney which we had observed before in angiotensin II-treated mice [8,11,17]. In contrast to the small kidney vessels, hypertrophy of the thoracic aorta as measured by Matsuno et al. was not reduced by the Nox1 knockout [25]. In the present study, damage parameters of glomeruli and tubuli were not changed in any of the angiotensin II-treated groups, unlike in a former study [8]. Morphometry showed the prevention of glomerular hypertrophy and mesangial expansion following Nox1 knockout [31], while knocking out Nox2 had no effect on diabetes-induced morphological changes [32].

Kidney function, as measured via creatinine clearance, was not altered by angiotensin II treatment. As a second and more sensitive parameter of kidney function, albumin concentration in urine was quantified. All our angiotensin II-treated animals had increased albumin values. In Nox1 knockout animals, this albuminuria was significantly lower, and in Nox4 knockout animals tendentially lower than in the wildtype animals. In diabetic animals, Nox2 knockout could not protect the kidney from developing albuminuria [32]. For Nox4, both possibilities, protection and lack of protection from albuminuria, have been reported for diabetic animals, which could be due to the use of different diabetes models (i.e., streptozotocin-induced versus ApoE knockout mice) [33,34]. Knocking out Nox1 also could not prevent albuminuria in diabetic animals [34] and no publications measuring albumin in nondiabetic angiotensin II-treated animals were found. Therefore, to the best of our knowledge, this is the first report demonstrating a reduction of albuminuria in Nox1 knockout animals in the setting of angiotensin II-induced hypertension.

With the exception of the Nox4 knockout mice, all angiotensin II-treated animals analyzed in the present study, exhibited significant cardiac hypertrophy, which is a usual outcome of hypertension [35]. Calculating the gain in heart weight, Nox2 knockout mice revealed the highest increase, followed by wildtype mice, Nox1 knockout mice, and Nox4 knockout mice as last. This ranking roughly reflects the increase in blood pressure, where the Nox4 and Nox1 knockout animals had the lowest increase in blood pressure.

NADPH oxidase activation is often associated with oxidative stress. Nox1 knockout reduces ROS levels in models of angiotensin II-induced hypertension (heart and kidney) [21,25], diabetes [31], and atherosclerosis [36]. For Nox1 and Nox4, reports can be found, which show a protective role of Nox1 or Nox4 function, since loss of the enzymes increased tissue damage; atherosclerosis in the absence of Nox1 or Nox4 [37,38] and tubulo-interstitial renal injury (in the unilateral ureter obstruction model, UUO) if Nox4 was missing [33]. A marker of systemic oxidative stress to lipids, 15-isoprostane F_2t_, was already much lower in the untreated Nox1 knockout animals as compared with the untreated wildtype animals. 15-isoprostane F2t did not increase significantly in the angiotensin II-treated Nox1 knockout mice, indicating a lower body burden of ROS when Nox1 was missing. Although, in the other knockout animals, a significant increase of 15-isoprostane F_2t_ by angiotensin II treatment also could not be detected, and their basal levels of 15-isoprostane F_2t_ were not lower than those of the control. Lower lipid peroxidation was also seen in Nox1 knockout mice with diabetes [31] and liver fibrosis [39]. In the present study, the residual increase of oxidative stress markers in angiotensin II-treated Nox1 knockout animals could be caused by other ROS generating enzymes that were not analyzed here.

Strikingly, lack of Nox2 and Nox4 led to a higher basal DNA damage in the kidney of the knockout animals. Higher basal levels of DNA damage were recently also observed in vitro, in Nox4-knockout cells [40]. In that study, a role of Nox4 in DNA damage repair was discussed; oxidation of the kinase AKT in the nucleus by Nox4 was necessary to keep the activity of the γ-H2AX-dephosphorylating phosphatase PP2A low [40]. Therefore, loss of Nox4 impaired DNA repair and elevated DNA damage [40]. The importance of Nox4 and Nox1 for phosphorylation of γ-H2AX was also shown by Kodama et al. [41], although DNA damage was not quantified in their study. In our study, induction of DNA damage by angiotensin II was only observed in the wildtype animals, and if the comet assay was used, it was also observed in the Nox4 knockout animals. Nox1 knockout animals did not show higher basal DNA damage in kidney cells and, moreover, also no increase by angiotensin II treatment. Rather, their values were even significantly lower than those of angiotensin II-treated wildtype animals. This is in line with the observation of reduced oxidative stress levels in this group. Nox1 was suggested to be involved in tumor progression, since knockout of this isoform reduced the size of fibro sarcomas, colon, and liver tumors [40,42,43]. As molecular mechanisms, the role of Nox1 in the production of inflammatory cytokines or in the promotion of angiogenesis has been discussed. There have also been data from humans showing that high Nox1 concentration is an independent predictor of shorter survival after treatment of hepatocellular carcinoma by hepatectomy [44]. Here, we suggest that it is also the role of Nox1 in DNA damage induction that contributes to adverse effects of Nox1 activation.

## 5. Conclusions

Angiotensin II-induced hypertension is accompanied by impairment of kidney function, increased oxidative stress, and higher levels of DNA damage to kidney cells. The NADPH oxidase isoform Nox1 was identified to play a major role in provoking oxidative stress and DNA damage after angiotensin II treatment. Furthermore, a so far unknown role of Nox1 in dipsogenic effects of angiotensin II was identified. Pharmacological targeting of Nox1 could be beneficial for slowing down the progression of hypertension-induced kidney damage.

## Figures and Tables

**Table 1:** Blood pressure, body weight, and body weight ratios of the animals.

		Body Weight (g)	Relative Kidney Weight (‰)	Relative Heart Weight (‰)	Systolic Blood Pressure (mmHg)	Diastolic Blood Pressure (mmHg)
Wildtype	Control	30.6 ± 0.3	6.4 ± 0.2	6.3 ± 0.2	127 ± 2	83 ± 3
	AngII	28.1 ± 0.3 ***	6.6 ± 0.2	7.9 ± 0.3 ***	194 ± 3 ***	121 ± 4 ***
Nox1^y/−^	Control	28.3 ± 0.6 ^++^	6.0 ±.0.2	6.3 ± 0.2	124 ± 2	84 ± 4
	AngII	27.9 ± 0.8	6.0 ± 0.1	7.3 ± 0.4 *	163 ± 5 ***^,^°°°	103 ± 9
Nox2^y/−^	Control	33.3 ± 0.9 ^+++^	6.2 ± 0.2	5.6 ± 0.2	123 ± 5	78 ± 9
	AngII	28.6 ± 0.6 ***	6.3 ± 0.2	7.2 ± 0.4 **	181 ± 6 ***	129 ± 8 ***
Nox4^−/−^	Control	31.1 ± 0.5	6.1 ± 0.2	6.5 ± 0.4	115 ± 4	70 ± 4
	AngII	27.7 ± 0.6 ***	6.0 ± 0.2	7.0 ± 0.5	170 ± 7 ***^,^°°°	118 ± 5 ***

* *p* ≤ 0.05, ** *p* < 0.01, *** *p* < 0.001 vs. the respective untreated animals (Student’s *t*-test); ^++^ *p* < 0.01 (F(1.49) = 9.99), ^+++^ *p* < 0.001 (F(1.46) = 52.60) vs. wildtype control; °°° *p* < 0.001 (FNox1(1.49) = 24.36, FNox4(1.54) = 25.16) vs. angiotensin II-treated wildtype (2-way ANOVA with Tukey’s multiple comparisons test). AngII = angiotensin II, Nox = NADPH oxidase catalytic subunit.

**Table 2:** Drink volume, creatinine clearance, and histological parameters.

		Drink Volume [mL]	Creatinine Clearance [µL/min]	GSI	MSI	TSI	VSI
Wildtype	Control	3.9 ± 0.3	73 ± 9	1.1 ± 0.1	1.0 ± 0.2	0.1 ± 0.2	0.7 ± 0.1
	AngII	7.1 ± 0.5 ***	74 ± 13	1.3 ± 0.1	1.1 ± 0.2	0.1 ± 0.1	1.1 ± 0.1 ***
Nox1^y/−^	Control	4.4 ± 0.7	94 ± 11	1.2 ± 0.1	0.3 ± 0.1 ^+^	0.1 ± 0.0	0.6 ± 0.1
	AngII	4.5 ± 1.1	81 ± 8	1.4 ± 0.2	0.4 ± 0.1	0.1 ± 0.1	0.8 ± 0.1 °
Nox2^y/−^	Control	3.5 ± 0.2	105 ± 22	1.2 ± 0.1	0.8 ± 0.1	0.3 ± 0.0	0.9 ± 0.1
	AngII	7.6 ± 0.8 ***	77 ± 7	1.0 ± 0.1	0.6 ± 0.0	0.2 ± 0.0	1.3 ± 0.1 *
Nox4^−/−^	Control	3.8 ± 0.4	53 ± 11	1.3 ± 0.1	1.2 ± 0.1	0.1 ± 0.0	0.8 ± 0.1
	AngII	7.4 ± 0.9 **	52 ± 5	1.2 ± 0.0	1.5 ± 0.1 **	0.1 ± 0.0	1.1 ± 0.1 *

* *p* ≤ 0.05, ** *p* < 0.01, *** *p* < 0.001 vs. the respective untreated animals (Student’s *t*-test); ^+^ *p* ≤ 0.05 (F(1.49) = 15.86) vs. wildtype control; ° *p* ≤ 0.05 (F(1.49) = 16.61) vs. angiotensin II-treated wildtype (2-way ANOVA). AngII, angiotensin II; Nox, NADPH oxidase catalytic subunit; GSI, glomerular sclerosis index; MSI, mesangiolysis index; TSI, tubulointerstitial sclerosis index; VSI, vascular sclerosis index.

## References

[1] Colt J.S., Schwartz K., Graubard B.I., Davis F., Ruterbusch J., DiGaetano R., Purdue M., Rothman N., Wacholder S., Chow W.H. (2011). Hypertension and risk of renal cell carcinoma among white and black Americans. Epidemiology.

[2] Flaherty K.T., Fuchs C.S., Colditz G.A., Stampfer M.J., Speizer F.E., Willett W.C., Curhan G.C. (2005). A prospective study of body mass index, hypertension, and smoking and the risk of renal cell carcinoma (United States). Cancer Causes Control.

[3] Hsieh J.J., Purdue M.P., Signoretti S., Swanton C., Albiges L., Schmidinger M., Heng D.Y., Larkin J., Ficarra V. (2017). Renal cell carcinoma. Nat. Rev. Dis. Primers.

[4] Laragh J.H., Lewis K. (1992). Dahl Memorial Lecture. The renin system and four lines fo hypertension research. Nephron heterogeneity, the calcium connection, the prorenin vasodilator limb, and plasma renin and heart attack. Hypertension.

[5] Deckers I.A., van den Brandt P.A., van Engeland M., van Schooten F.J., Godschalk R.W., Keszei A.P., Schouten L.J. (2015). Polymorphisms in genes of the renin-angiotensin-aldosterone system and renal cell cancer risk: Interplay with hypertension and intakes of sodium, potassium and fluid. Int. J. Cancer.

[6] Schupp N., Schmid U., Rutkowski P., Lakner U., Kanase N., Heidland A., Stopper H. (2007). Angiotensin II-induced genomic damage in renal cells can be prevented by angiotensin II type 1 receptor blockage or radical scavenging. Am. J. Physiol. Renal Physiol..

[7] Schmid U., Stopper H., Schweda F., Queisser N., Schupp N. (2008). Angiotensin II induces DNA damage in the kidney. Cancer Res..

[8] Brand S., Amann K., Schupp N. (2013). Angiotensin II-induced hypertension dose-dependently leads to oxidative stress and DNA damage in mouse kidneys and hearts. J. Hypertens..

[9] Hartmann C., Schulz I., Epe B., Schupp N. (2019). Angiotensin II-induced hypertension increases the mutant frequency in rat kidney. Arch. Toxicol..

[10] Fazeli G., Stopper H., Schinzel R., Ni C.W., Jo H., Schupp N. (2012). Angiotensin II induces DNA damage via AT1 receptor and NADPH oxidase isoform Nox4. Mutagenesis.

[11] Brand S., Amann K., Mandel P., Zimnol A., Schupp N. (2014). Oxidative DNA damage in kidneys and heart of hypertensive mice is prevented by blocking angiotensin II and aldosterone receptors. PLoS ONE.

[12] Garrido A.M., Griendling K.K. (2009). NADPH oxidases and angiotensin II receptor signaling. Mol. Cell Endocrinol..

[13] Santillo M., Colantuoni A., Mondola P., Guida B., Damiano S. (2015). NOX signaling in molecular cardiovascular mechanisms involved in the blood pressure homeostasis. Front. Physiol..

[14] Bedard K., Krause K.H. (2007). The NOX family of ROS-generating NADPH oxidases: Physiology and pathophysiology. Physiol. Rev..

[15] Gill P.S., Wilcox C.S. (2006). NADPH oxidases in the kidney. Antioxid. Redox Signal..

[16] Tu H., Wen C.P., Tsai S.P., Chow W.H., Wen C., Ye Y., Zhao H., Tsai M.K., Huang M., Dinney C.P. (2018). Cancer risk associated with chronic diseases and disease markers: Prospective cohort study. BMJ.

[17] Zimnol A., Amann K., Mandel P., Hartmann C., Schupp N. (2017). Angiotensin II type 1a receptor-deficient mice develop angiotensin II-induced oxidative stress and DNA damage without blood pressure increase. Am. J. Physiol. Renal Physiol..

[18] Westhoff J.H., Hilgers K.F., Steinbach M.P., Hartner A., Klanke B., Amann K., Melk A. (2008). Hypertension induces somatic cellular senescence in rats and humans by induction of cell cycle inhibitor p16INK4a. Hypertension.

[19] Schupp N., Kolkhof P., Queisser N., Gärtner S., Schmid U., Kretschmer A., Hartmann E., Oli R.G., Schäfer S., Stopper H. (2011). Mineralocorticoid receptor-mediated DNA damage in kidneys of DOCA-salt hypertensive rats. FASEB J..

[20] Lamprecht M.R., Sabatini D.M., Carpenter A.E. (2007). CellProfiler: Free, versatile software for automated biological image analysis. Biotechniques.

[21] Yogi A., Mercure C., Touyz J., Callera G.E., Montezano A.C., Aranha A.B., Tostes R.C., Reudelhuber T., Touyz R.M. (2008). Renal redox-sensitive signaling, but not blood pressure, is attenuated by Nox1 knockout in angiotensin II-dependent chronic hypertension. Hypertension.

[22] Chrissobolis S., Banfi B., Sobey C.G., Faraci F.M. (2012). Role of Nox isoforms in angiotensin II-induced oxidative stress and endothelial dysfunction in brain. J. Appl. Physiol..

[23] Johar S., Cave A.C., Narayanapanicker A., Grieve D.J., Shah A.M. (2006). Aldosterone mediates angiotensin II-induced interstitial cardiac fibrosis via a Nox2-containing NADPH oxidase. FASEB J..

[24] Schroder K., Zhang M., Benkhoff S., Mieth A., Pliquett R., Kosowski J., Kruse C., Luedike P., Michaelis U.R., Weissmann N. (2012). Nox4 is a protective reactive oxygen species generating vascular NADPH oxidase. Circ. Res..

[25] Matsuno K., Yamada H., Iwata K., Jin D., Katsuyama M., Matsuki M., Takai S., Yamanishi K., Miyazaki M., Matsubara H. (2005). Nox1 is involved in angiotensin II-mediated hypertension: A study in Nox1-deficient mice. Circulation.

[26] Byrne J.A., Grieve D.J., Bendall J.K., Li J.M., Gove C., Lambeth J.D., Cave A.C., Shah A.M. (2003). Contrasting roles of NADPH oxidase isoforms in pressure-overload versus angiotensin II-induced cardiac hypertrophy. Circ. Res..

[27] Bouabout G., Ayme-Dietrich E., Jacob H., Champy M.F., Birling M.C., Pavlovic G., Madeira L., Fertak L.E., Petit-Demouliere B., Sorg T. (2018). Nox4 genetic inhibition in experimental hypertension and metabolic syndrome. Arch. Cardiovasc. Dis..

[28] Miyano K., Sumimoto H. (2007). Role of the small GTPase Rac in p22phox-dependent NADPH oxidases. Biochimie.

[29] Zimmerman M.C., Dunlay R.P., Lazartigues E., Zhang Y., Sharma R.V., Engelhardt J.F., Davisson R.L. (2004). Requirement for Rac1-dependent NADPH oxidase in the cardiovascular and dipsogenic actions of angiotensin II in the brain. Circ. Res..

[30] Peterson J.R., Burmeister M.A., Tian X., Zhou Y., Guruju M.R., Stupinski J.A., Sharma R.V., Davisson R.L. (2009). Genetic silencing of Nox2 and Nox4 reveals differential roles of these NADPH oxidase homologues in the vasopressor and dipsogenic effects of brain angiotensin II. Hypertension.

[31] Zhu K., Kakehi T., Matsumoto M., Iwata K., Ibi M., Ohshima Y., Zhang J., Liu J., Wen X., Taye A. (2015). NADPH oxidase NOX1 is involved in activation of protein kinase C and premature senescence in early stage diabetic kidney. Free Radic. Biol. Med..

[32] You Y.H., Okada S., Ly S., Jandeleit-Dahm K., Barit D., Namikoshi T., Sharma K. (2013). Role of Nox2 in diabetic kidney disease. Am. J. Physiol. Renal Physiol..

[33] Babelova A., Avaniadi D., Jung O., Fork C., Beckmann J., Kosowski J., Weissmann N., Anilkumar N., Shah A.M., Schaefer L. (2012). Role of Nox4 in murine models of kidney disease. Free Radic. Biol. Med..

[34] Jha J.C., Gray S.P., Barit D., Okabe J., El-Osta A., Namikoshi T., Thallas-Bonke V., Wingler K., Szyndralewiez C., Heitz F. (2014). Genetic targeting or pharmacologic inhibition of NADPH oxidase nox4 provides renoprotection in long-term diabetic nephropathy. J. Am. Soc. Nephrol..

[35] Nwabuo C.C., Vasan R.S. (2020). Pathophysiology of Hypertensive Heart Disease: Beyond Left Ventricular Hypertrophy. Curr. Hypertens. Rep..

[36] Di Marco E., Gray S.P., Chew P., Kennedy K., Cooper M.E., Schmidt H.H., Jandeleit-Dahm K.A. (2016). Differential effects of NOX4 and NOX1 on immune cell-mediated inflammation in the aortic sinus of diabetic ApoE-/-mice. Clin. Sci..

[37] Sobey C.G., Judkins C.P., Rivera J., Lewis C.V., Diep H., Lee H.W., Kemp-Harper B.K., Broughton B.R., Selemidis S., Gaspari T.A. (2015). NOX1 deficiency in apolipoprotein E-knockout mice is associated with elevated plasma lipids and enhanced atherosclerosis. Free Radic. Res..

[38] Gray S.P., Di Marco E., Kennedy K., Chew P., Okabe J., El-Osta A., Calkin A.C., Biessen E.A., Touyz R.M., Cooper M.E. (2016). Reactive Oxygen Species Can Provide Atheroprotection via NOX4-Dependent Inhibition of Inflammation and Vascular Remodeling. Arterioscler. Thromb. Vasc. Biol..

[39] Lan T., Kisseleva T., Brenner D.A. (2015). Deficiency of NOX1 or NOX4 Prevents Liver Inflammation and Fibrosis in Mice through Inhibition of Hepatic Stellate Cell Activation. PLoS ONE.

[40] Helfinger V.V.G.F., Henke N., Kunze M.M., Schmid T., Heidler J., Wittig I., Radeke H.H., Marschall V., Anderson K. (2017). Hydrogen peroxide formation by Nox4 limits malignant transformation. bioRxiv.

[41] Kodama R., Kato M., Furuta S., Ueno S., Zhang Y., Matsuno K., Yabe-Nishimura C., Tanaka E., Kamata T. (2013). ROS-generating oxidases Nox1 and Nox4 contribute to oncogenic Ras-induced premature senescence. Genes Cells.

[42] Stalin J., Garrido-Urbani S., Heitz F., Szyndralewiez C., Jemelin S., Coquoz O., Ruegg C., Imhof B.A. (2019). Inhibition of host NOX1 blocks tumor growth and enhances checkpoint inhibitor-based immunotherapy. Life Sci. Alliance.

[43] Liang S., Ma H.Y., Zhong Z., Dhar D., Liu X., Xu J., Koyama Y., Nishio T., Karin D., Karin G. (2019). NADPH Oxidase 1 in Liver Macrophages Promotes Inflammation and Tumor Development in Mice. Gastroenterology.

[44] Ha S.Y., Paik Y.H., Yang J.W., Lee M.J., Bae H., Park C.K. (2016). NADPH Oxidase 1 and NADPH Oxidase 4 Have Opposite Prognostic Effects for Patients with Hepatocellular Carcinoma after Hepatectomy. Gut Liver.

